# Seroconversion and safety of Covid-19 vaccines in pa-tients with chronic liver disease and liver transplant: A systematic review

**DOI:** 10.5339/qmj.2023.21

**Published:** 2023-12-12

**Authors:** Abdel-Naser Y. Elzouki, Mohamed Nabil Elshafei, Afia Aziz, Islam Elzouki, Muhammad Aamir Waheed, Khalid Farooqui, Aftab Mohammed Azad, Elmukhtar Habas, Mo-hammed I. Danjuma

**Affiliations:** ^1^Department of Medicine, Hamad Medical Corporation, Doha, Qatar E-mail: aelzouki@hamad.qa ORCID ID: 000-0002-9132-1752; ^2^College of Medicine, Qatar University, Doha, Qatar; ^3^Weill Cornell Medicine-Qatar, Doha, Qatar; ^4^Department of Clinical Pharmacy, Hamad General Hospital, Hamad Medical Corporation, Doha, Qatar; ^5^Department of Emergency Medicine, Hamad General Hospital, Hamad Medical Corporation, Doha, Qatar

**Keywords:** Chronic liver disease, liver transplantation, Covid-19 vaccine, seroconversion response, efficacy, safety

## Abstract

Background and Aims: As part of the COVID-19 control strategy, a growing number of vaccine portfolios evolved and got fast-tracked through regulatory agencies, with a limited examination of their efficacy and safety in vulnerable populations, such as patients with chronic conditions and immunocompromised states. Patients with chronic liver disease (CLD), and cohorts post liver transplant (LT) in particular, were underrepresented in the determinant trials of vaccine development, hence the paucity of data on their efficacy and safety in published literature. This systematic review aims to examine the available evidence and ascertain the effectiveness and safety of Covid-19 vaccination in patients with CLD and those with LT.

Methods: A systematic review of PubMed (Medline), Google Scholar, Cochrane Library, and ScienceDirect from inception until 1^st^ March 2022 was conducted. We included observational studies and assessed vaccine efficacy regarding seroconversion or immunological rate, whereas serious or significant adverse effects have been considered safety outcomes when reported.

Results: Studies comprised 45275 patients, performed in 11 different countries. Seroconversion or immunological rate after Covid-19 vaccination was mostly the primary endpoint, whereas other endpoints like covid-19 related adverse effects were also reported. Twenty-four of the final analyzed studies are prospective cohort studies, while four are retrospective cohort studies. Twenty-one studies included patients who underwent LT and received the Covid vaccine; nine included patients who had CLD due to various etiologies. The median age range of all included patients varied from 43–69 years. All patients with LT who received at least two doses of Covid vaccine had a seroconversion rate of around 60%. Patients with CLD had a seroconversion rate of about 92% post two doses of Covid vaccination. The average seroconversion rate in post-transplant recipients was around 45% after two doses of the significant Covid vaccines: Pfizer, AstraZeneca, Moderna, and Jansen. Only two studies have reported a higher seroconversion rate of 75% and 73% after the third dose of Covid vaccine. No significant adverse effects were reported in all studies; the most commonly reported negative effect was local injection site pain.

Conclusion: The present systematic review, comprising real-world observational data studies, concludes that Covid-19 vaccination was associated with 92% and 60% seroconversion rates in patients with CLD and LT, respectively. No significant side effects were reported in all studies. This finding helps to resolve the uncertainty associated with Covid-19 vaccination in this cohort of patients.

## Introduction

The first case of Coronavirus disease 2019 (Covid-19) was identified in Wuhan, China, on December 2019.^1^ It quickly escalated to engulf the world and become a pandemic affecting every human at every phase of life, impacting politics, economics, and social policies. The beginning of the Covid-19 pandemic also led to a new era of realization that technological advancements had made it possible to create a vaccine in a year. However, it gave rise to numerous questions along the way. While due importance was given to the development of covid-19 vaccines at a rapid pace, most of these covid-19 vaccines were approved for emergency use without the data of their efficacy and side-effect profile in patients with various co-morbidities and chronic disorders, including patients with cirrhosis, chronic kidney disease, cardiovascular and chronic respiratory diseases as well as immunosuppression as these trials excluded patients with chronic conditions and immunosuppression and focused only on healthy populations.^2^

However, it was also noted that this group of patients was susceptible to covid-19 complications that led to increased mortality.^3,4^ Indeed, Schinas et al., and Sripongpun et al., while acknowledging the inherent risks of additional therapeutic intervention (by way of immunization), pushed forward recommendations for COVID-19 vaccination in this cohort of patients. They acknowledge society guidelines recommendations primarily drove this at the mid-point of the pandemic, but lately, from primary trial and real-world vaccine response and safety data of patients with LT and CLD.^5,6^ Only a limited number of patients with liver disease were studied in BNT162b2 mRNA Covid-19 Vaccine (Pfizer) Phase 3 trials (217 of 37706 patients or 0.6%) and even fewer in mRNA-1273 SARS-CoV-2 Vaccine (Moderna) Phase 3 trials [196 chronic liver disease (CLD) patients out of 30351 (0.7%)].^7,8^ 1.5% of patients enrolled for AstraZeneca studies had liver disease.^9^ Conversely, BBIBP-CorV (Sinopharm) inactivated SARS-CoV2 vaccine excluded immunocompromised (solid organ transplant patients) and chronic liver disease patients.^10^ With the roll-out of vaccines for emergency use, efficacy, seroprevalence, and immunogenicity in cirrhosis and liver transplant patients, and patients asking the relevant question of how safe and effective this vaccine is for them becomes the million-dollar question. Emerging data on reduced seroprevalence rates in various solid organ transplant recipients has been observed, and questions regarding a need for third dosing have also been raised.^11,12^

Various associations have given their recommendations, including the American Association for the Study of Liver Disease (AASLD) and the European Association of Liver Disease (EASL), with regards to vaccine administration and encouraged patients to be vaccinated as the benefits outweigh the harm with the risk of infection-related complications and increased risk of mortality.^13–15^ Before the onset of the pandemic, clinical risk prognostication scores existed, including one of the most validated, called the Model for End-stage Liver Disease (MELD) score.^16^ The latter is a prospectively developed and validated cirrhosis severity scoring system that uses a patient’s laboratory values for serum bilirubin, serum creatinine, and the international normalized ratio (INR) to predict three-month survival. In patients with cirrhosis, an increasing MELD score is associated with increasing severity of hepatic dysfunction and three-month mortality risk. A modified MELD score, called MELD-Na, recently became the standard for organ allocation for liver transplantation (LT); MELD-Na score takes into account Na values of 125–137.^17^

Covid-19 results in a strong humoral and cellular immune response.^18^ Antibodies (IgG and IgM) are classically detected in the second week of symptom onset, with IgM antibody appearing first and IgG being the most durable. Among the Covid-19 structural proteins, the Spike (S) and the Nucleocapsid (N) proteins are the main immunogens.^19^ The S protein consists of two subunits, S1, which contains the Receptor Binding Domain (RBD) and S2. Most available vaccines include only a fragment of the spike protein and induce antibodies to S, particularly the RBD on the S1 subunit of the spike protein.^20^ The Anti-RBD (or Spike) antibodies prevent the virus from penetrating the cells by inhibiting its binding to the Angiotensin-Converting Enzyme-2 (ACE-2) receptor in the epithelial cells of several human organs and tissues. Cellular immune responses against the virus, although considered essential to control the infection, are not commercially available but have been used for research purposes.

Kulkarni et al.’s systematic review observed increasing Covid-related mortality, especially in liver transplant (LT) patients.^21^ It was also observed that cirrhotic patients’ especially those with acute-on-chronic liver disease and those with higher Child class and MELD scores as well as high serum creatinine levels, had higher mortality rates due to Covid-19.^3^ Therefore, vaccinating these individuals is of paramount importance. But data regarding CLD and the effect of vaccinations on these patients remains scarce, and the question remains unanswered related to vaccine efficacy and side-effects concerns. Therefore, we aimed to conduct a systematic review based on already available data to determine if the efficacy and side-effect profile of Covid-19 vaccines on these particular patients’ populations could be established.

## Methods

### Research question addressed

This systematic review was performed according to PRISMA 2021 guidelines.^22^ The systematic review protocol was formed before undertaking the data collection but needed to be registered. It is linked in the appendix. The research questions were: What is the effect of Covid-19 vaccines (mRNA, inactivated virion, viral vector, protein subunit vaccines) on efficacy, immunogenicity, and side effects in CLD and LT patients?

### Eligibility criteria

We used the population-intervention-comparator-outcomes (PICO) framework to establish the eligibility criteria.

#### Population

Patients with CLD, including (chronic hepatitis B, hepatitis C, Autoimmune hepatitis, nonalcoholic fatty liver disease, Alcohol-induced hepatitis, hemochromatosis, cirrhosis, and liver transplant).

#### Intervention

At least one dose of Covid-19 vaccine intake (mRNA, inactivated virion, viral vector, protein subunit vaccines).

#### Outcome

Efficacy and immunogenicity with antibodies development and reported adverse effects of Covid-19 vaccines in CLD and LT patients. We included all studies regardless of the bioanalytical methods for the determination of immunogenicity (such as Enzyme-Linked Immunoassay (ELISA), antibody assays, Chemiluminescence immunoassay [CLIA], Electrochemiluminescence immunoassay [ECLIA], etc.).

#### Inclusion and exclusion

We have included all prospective and retrospective studies in patients with CLD or liver transplantation except case reports and case series. References from various studies were also reviewed. All studies in English were included as the reviewer’s primary language of communication. All case reports, case series and analyses in languages other than English were excluded. All studies with healthy patients and animal studies were excluded.

### Search strategy

Two reviewers independently performed a comprehensive search (A.A and M.E). PubMed (Medline), Google Scholar, Cochrane Library, and ScienceDirect included databases. We integrated controlled vocabulary (e.g., “Covid-19 Vaccines”[All Fields] OR “COVID-19 Vaccines”[MeSH Terms] AND liver disease OR cirrhosis OR liver transplant) and keywords (e.g., Covid-19 vaccines and CLD or cirrhosis or LT) into the searches. The comprehensive search strategy is included in the appendices for reference. Limits and filters used were language restriction to English language only and date restriction to 1^st^ January 2020 till 1^st^ March 2022. We also performed a manual reference search from pre-existing and retrieved studies.

### Screening

**Figure 1.**
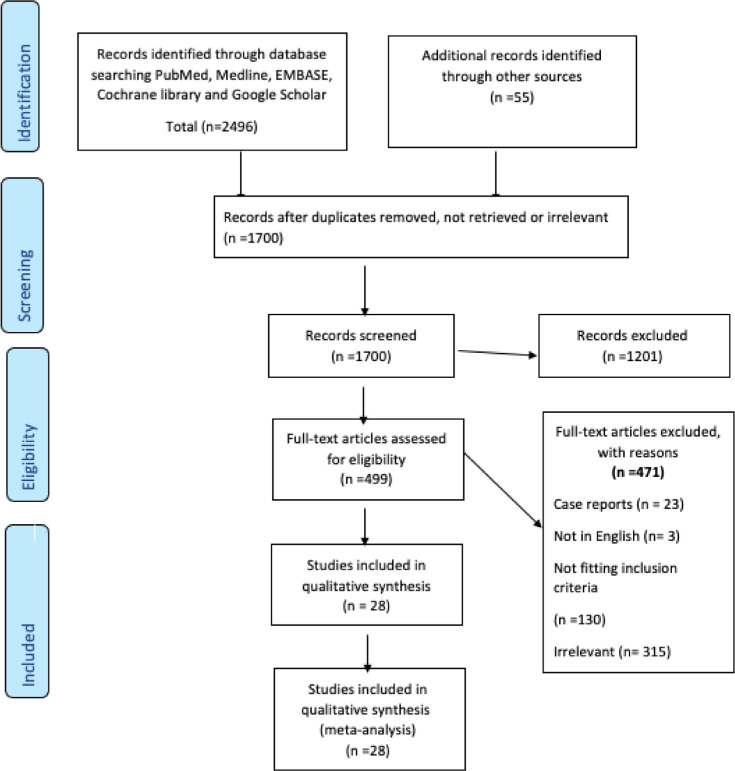
PRISMA flow diagram.

The screening was done in two stages, with an initial title and abstract screening attempted by two reviewers independently (A.A and M.E). Articles were eligible for full-text review if they included any terms related to covid-19 vaccines and liver disease or liver transplantation. Eligible papers were then evaluated for inclusion; any discrepancy in the study selection was clarified by mutual discussion, and if still not settled by discussion, a third reviewer (A-EZ) resolved the disagreement as guided by the protocol. The PRISMA flow diagram shown in Figure 1 depicts our search methodology.

### Quality appraisal with risk of bias assessment

Quality appraisal was done with standardized tools, namely Newcastle Ottawa Scale tool, for assessing the quality and risk of bias in cross-sectional and cohort studies. Quality appraisal of studies included in data synthesis is shown in Table 1.

### Data extraction

Data extraction was performed utilizing preplanned templates. Data extracted included author, year of publication, study design, country, median age, patient population, control, outcome assessed, type of vaccine, and significant adverse effects.

### Data analysis

Descriptive statistics such as median and interquartile range were calculated for age and sample size; frequencies (percentages) were reported for categorical data. All analyses were conducted with Stata (StataCorp. 2015. Stata Statistical Software: Release 14. College Station, TX: StataCorp LP).

## Results

Our screening initially revealed 2496 titles. After removal of duplicates, reviews, and meta-analysis, it yielded 1645, which were screened. After screening, 28 studies were included in our final review (Figure 1 shows the PRISMA flow diagram). Our literature review yielded 27 studies.^3,22–47^ Four studies were retrospective, while the rest were prospective studies Table 1).

Studies comprised 45275 patients considered in our review; patients included in our review either have CLD (cirrhosis) or post-liver transplantation. Analyses were performed in 11 different countries, and the median age range of all included patients varied from 43 to 69 years. Seroconversion or immunological rate after Covid-19 vaccination was mostly the primary endpoint, whereas other endpoints like Covid-19-related adverse effects were also reported. Twenty-one studies included patients who underwent LT and received the Covid-19 vaccine; the rest had CLD due to various etiologies. Table 1 summarizes the CLD and LT patients’ characteristics, study designs, type of used vaccines, serological response rates, and significant adverse events of vaccines. All patients with LT who received at least two doses of the Covid-19 vaccine had a seroconversion rate of around 60%. Patients with CLD had a seroconversion rate of about 92% post two doses of Covid-19 vaccination. Two Del Bello et al.^23^ and Kamar et al.^31^ reported a higher seroconversion rate of 73% and 75%, respectively, after the third dose of the Covid-19 vaccine. No significant adverse effects were reported in all studies; the most reported adverse effects were local injection site pain; however, no derangements of liver function tests or liver-related complications were reported. Table 2 shows the status of Covid-19 infection exposure, etiology of CLD, indication for LT, and types of antibodies measured in the population of the included studies.

## Discussion

The finding of this review represents the first comprehensive examination of published reports on the effect of Covid-19 immunization in patients with CLD patients with cirrhosis. Our qualitative synthesis of *n* = 45275 patients amongst^28^ studies found that patients with CLD had seroconversion rates of about 92% after receiving two doses of Covid-19 vaccination. A subset of those with LT had a comparatively lower seroconversion rate of approximately 60%. From previous reports, patients with CLD have suboptimal innate and humoral-mediated responses to infective agents, making a seroconversion rate of 92%; therefore, both are reassuring, especially in the absence of significant vaccine-related adverse events. Constituents’ studies in our synthesis have reported variable seroconversion proportions but with uniform rates amongst patients with CLD.^49^ There was no difference in seroconversion response among cirrhotic and non-cirrhotic groups.^49^ This undoubtedly will have tremendous implications regarding the need and immunization schedules of these vulnerable cohorts of patients. Studies thus far involving various Covid-19 patient cohorts have consistently demonstrated a poorer outcome for those with liver diseases of varying primary etiologies compared to those without.^50^ With the onset of the Covid-19 pandemic, several SARS-COV vaccines have been developed at a geometric pace, with subsequent review and multi-level, and sometimes patient-specific approval by regulatory agencies worldwide (including the FDA). These include Pfizer/BioNTech BNT162b2 mRNA^7^ Moderna mRNA-1273,^8^ and the AstraZeneca/University of Oxford ChAdOx1-nCoV-19 chimpanzee adenovirus (ChAd).^9^ Although these vaccines reported excellent efficacy and safety profiles in healthy individuals, uncertainty remains regarding their effectiveness and safety in patients’ cohorts with CLD. This arose from the paucity of available data and the small sample size of the few studies exploring this. For example, despite thousands of patients enrolled in these trials, there is conspicuous underrepresentation of the proportion of those with CLD. In the Pfizer vaccination study, for example, 217 (0·6%) of 37706 participants had liver disease, and only three (<0·1%) had moderate to severe liver disease. A similarly low proportion of patients with CLD were included in the Moderna trial (196 of 30351 patients [0·6%]). Indeed, the ChAdOx1-nCoV-19 vaccine trial excluded patients with pre-existing liver disease.

Our finding of comparatively lower seroconversion rates (60%) amongst patients with LT was rather interesting. This observation appears consistent with those of Thuluvath et al.,^49^, who reported a failed response rate following Johnson and Johnson vaccination of 61% and 24% for liver transplant recipients and those with CLD, respectively. Compared to other solid organ transplants (such as patients with Kidney transplants), our point estimate of seroconversion rates appears satisfactory, with 19% to 25% reported in some studies.^51^ Pathophysiologically, this could be attributable to the pervading induced immune tolerance obligated by the many immunosuppressive pharmacological agents these patients often have to take.^31,47^ A combination of other factors, including disordered inflammatory response and tardiness of B-cell response, are expected to impact the immunogenicity of administered vaccines negatively. Reassuringly both our review and recently published reports have all suggested otherwise. It is reassuring that these points estimate lies within the ballpark reported in other related morbidities characterized by underlying disease-related or pharmacologically induced immune modulation and exposure to COVID-19 vaccination.

The overall pooled point estimate of seroconversion response from our review falls within the reported estimate of 47.5% after two doses of the COVID-19 vaccine (from some studies). Data from studies examining the natural history of COVID-19 and those exploring interventions and biological prevention (such as immunizations) have consistently reported poorer outcomes amongst patient cohorts with multi-morbidity.^3^ Increasing long-term condition counts are often associated with multiplicative risks of adverse COVID-19 outcomes.^3^ However, we did not observe any significant difference in response rate based on comorbidities. While this may be reassuring, it may require further exploration by future studies. Similarly, we found no differences in estimates of seroconversion percentages amongst patients receiving different types of COVID-19 vaccines. This is hardly surprising, given the uniformity of efficacy of the various vaccines despite differences in the constituent products and as well as formulations.^52^

Since the onset of the COVID-19 pandemic, earlier and subsequent studies have not shown any causal relationship between COVID-19 vaccination and hepatic injury.^53^ This mirrors our observation in these pooled analyses. Similarly, the proportion of patients with adverse events following vaccination was negligible, consistent with previous reports, including those by Tu et al.^54^ In common with earlier reports, we found fatigue and dizziness remained the most reported adverse effects following COVID-19 vaccination.

### Strength and limitations

Our review represents the first and the most comprehensive pooled examination of the estimates of seroconversion rates amongst patients with CLD and LT following COVID-19 immunization. Our finding of differential point estimates amongst these two populations will undoubtedly be both reassuring and provide a supporting level of evidence for consideration of booster vaccine doses in the later cohort of patients. Our observation of excellent safety profiles across vaccine formulations in this hitherto pharmacologically vulnerable population is reassuring.

Consistent with previous reviews examining this variability in transplant patients, we encountered several limitations in this synthesis, including differences in the design of constituent studies, differences in the bioanalytical determination of antibody response, and the underlying etiology of primary liver disease, amongst others.^51^ Similarly, the absence of a weighted examination of the individual estimates of the constituent studies by way of meta-analysis meant that our results needed to be interpreted in this context. Additionally, examination of secondary data such as ours is often constrained by limitations of the constituent studies, which would have been minimized if we had proceeded to a meta-analysis (not possible because of evident heterogeneity). This notwithstanding, the overall findings of this systematic review favor vaccination of CLD patients and patients with liver transplants, emphasizing monitoring of the serological response after two doses of vaccine in patients post-LT. This is to ascertain the need for a booster dose in the event of the appearance of markers’ suboptimal response.

### Future perspectives

The result of this review has implications for both policy formulation at multiple levels and practice going forward. Healthcare providers and clinicians, in particular, could exploit the relatively favorable efficacy and safety profiles expounded by our synthesis in making informed decisions regarding Covid-19 vaccination in patients with LT and CLD. This is more so in settings where doubts and tardiness regarding recommendations for COVID-19 vaccination for eligible LT and CLD patients remain prevalent. Additionally, estimates reported from our synthesis will support other known estimates from further analyses in commissioning vaccination programs, including less tendency to exclude LT and CLD patients (due to perceived risks).

## Conclusions

A systematic examination of real-world observational studies found that Covid-19 vaccination was associated with 60% and 92% seroconversion rates in patients with LT and CLD, respectively. We found that no significant adverse effects were reported in all studies. This finding will help resolve the uncertainty regarding the efficacy and safety of Covid-19 vaccination in these cohorts of patients but also shows the need for further studies on the long-term phase IV safety of these vaccines in these vulnerable patient cohorts.

## Funding

No funding was sought for the conduct of this review.

## Ethical Declarations

No ethical approval is necessary as this was a secondary synthesis of published articles.

## Conflict of Interest

The authors declared no conflicts of interest relevant to this review or its publication.

## Research Involving Human and Animal Participants

Consent does not apply to this review. However, all authors consented and approved the final manuscript for publication.

## Figures and Tables

**Table 1.** Characteristics of the studies included in this review.

Author	Study design	Median Age (Year±SD or IQR)(Male %)	Patients (Received vaccine) (N)	Controls	Type of vaccine	Primary outcome	Major adverse events
**Studies Focusing on Liver Transplant Patients Only**							
Del Bello et al.^23^ France, 2021	Prospective	LT recipients 59 ± 15, (64.6%)	LT recipients (N=69) (cohort of 396 SOT recipients	No control	Pfizer-BioNTech BNT162b2	Positive Serological response four weeks after 3rd dose: 51 (73%)	Not reported
Boyarsky et al.^24^ USA, 2021	Prospective	55.9 [IQR, 41.3–67.4], (39%)	LT recipients (N=78) Liver transplant (LT) recipients (N=129) (Cohort of 658 SOT recipients)	No control	The first dose of: Pfizer BNT162b2 mRNA. OR Moderna mRNA-1273 Two doses of Pfizer BNT162b2 mRNA, or Moderna mRNA-1273	Humoral or cellular response LT patients: Detectable: 28 (37%) Non detectable: 50 (14%) Positive antibody response post 2 doses: 103(79%)	Not reported
Cholankeril et al.^25^ USA, 2022	Prospective	Cases 63(51–68) (70)	LT recipients (N=69)	No control	Two doses of Pfizer-BioNTech BNT162b2	Positive Anti-spike Antibody: 48% (n = 33) After 4 to 6 weeks of 2nd dose	Not reported
Erol et al.^26^ Turkey, 2021	Retrospective	Cases: 36.5 [18–62], (72.7%) Control: 37.5, NS	LT Patients N=10 (cohort of 48 SOT patients)	Health Care Workers (N=56)	Two doses of Pfizer-BioNTech BNT162b2 or Sinovac	100% seroconversion rates (6 received BNT162b2 Pfizer-BioNTech, and 4 Sinovac vaccine)	No severe adverse reactions were observed after either vaccine
D’Offizi et al.^27^ Italy, 2021	Prospective study	LT recipient 59 years [IQR 56–61], (70%). Healthy control (HC) group 43 years [IQR 36–53], (25.5%).	LT recipients (N=61)	Healthy control group (N=51)	BNT162b2 vaccine mRNA-1273 vaccine	Anti-spike response at T2 was significantly lower in LTRs compared with HCs (77.0% vs. 100%, P=0.001). N-Antibody response at T2 was observed in 29 LT recipients (47.5%) vs. 51 healthy controls (100%; P=0.001)	Not reported
Guarino et al.^28^ Italy, 2021	Prospective	LT recipients 62.52 ± 12.97, (76.4%) Control 57.86 ± 8.28. (64%)	LT recipients (N= 365)	Healthy healthcare workers N= 340	Pfizer-BioNTech BNT162b2 SARS-CoV-2	Serological response: LT: 273 (72.8%) Control: 335 (98.5%)	No major adverse events occurred in any of the enrolled patients
Hall et al.^29^ Canada, 2021	Prospective	SOT patients 66.2(IQR: 63.4–70.6), 69.3%	LT recipients (N= 15) (Cohort of 127 SOT recipients)	No control	Moderna mRNA-1273	Seroconversion rates after 2 doses: 8 (21.1%)	Local events most common were pain and swelling, while systemic events were fatigue, myalgias, and headache. No Grade 3 side effects noted
Herrera et al.^30^ Spain, 2021	Prospective	61.5(18–88) 69%	LT recipients (N=58)	No control	2 doses: Moderna COVID-19 mRNA	Humoral or cellular response. LT patients: After 1ST dose: 22 (37.9%) After 2nd dose: 41 (71%)	No change in transaminases, No rejection
Kamar et al.^31^ France, 2021	Prospective	LT recipients 58±2, (69%)	LT recipients (N=12) (Cohort of 101 SOT recipients)	No control	Pfizer-BioNTech BNT162b2	Positive Serological response After 3rd dose in LT patients: 9 (75%)	No serious adverse effects reported.
Marion et al.^32^ France, 2021	Prospective	LT recipients 59 ±1, (63.2%)	LT recipients (N=58)	No control	Pfizer-BioNTech BNT162b2 Moderna mRNA-1273	Positive Serological response: LT: 29 (50%)	Paresthesia of lower limb reported in one patient. No other reported side effects
Mazzola et al.^33^ France, 2022	Prospective	Cases LT recipients 61.0 (IQR: 55.0–67.0), 71.3% Control 55.0 (IQR: 38.0–62.0), 28%	LT recipients (N= 58)	Healthcare workers (N= 25)	BNT162b2 vaccine	Serological response: Liver transplant: 21(37.5%) Control 25 (100%)	No serious adverse events were reported
Nazaruk et al.^34^ Poland, 2021	Retrospective cohort study	LT recipients 58.4 (13.3), (N=80) Kidney -transplant recipients 54.4 (12.9), (45.9)	LT recipients (N= 55)	Kidney-transplant recipients (N=61)	BNT162b2 vaccine	The level of protective antibodies was 57.1% in kidney transplant recipients and 88.9% in LT recipients after the second dose.	Rejection episodes did not occur after vaccination.
Rabinowich et al.^35^ Israel, 2021	Prospective	Cases 60.1±12.8 (70) Control 63.22 ± 1\1.9 (66.7)	LT recipients (N =80)	Healthy individuals (N= 25)	Pfizer-BioNTech BNT162b2	Serological response Cases: 38(47%) Control: 100%	No events suspected or confirmed graft rejection, compromised graft function, neurological events or severe allergic reaction.
Rahav et al.^36^ Israel, 2021	Prospective cohort study	Cases: 68[51–71], (52.8%) Controls	LT Patients N=36	Healthy controls N=272	Two doses of the BNT162b2 mRNA vaccine	25/36(69.4%) developed anti-RBD antibodies after two to four weeks of the second vaccine dose	Vaccine-related serious AEs were not observed in the study
Rashidi-Alavijeh et al.^37^ Germany, 2021	Prospective	Cases 57 (IQR,49–64) (60.5) Control 43.5 (38–53.5) (45)	LT recipients (N= 43)	Healthcare workers (N = 20)	Pfizer-BioNTech BNT162b2	Immunogenicity rate LT recipients: 34 (79%) Control group (100%)	No episodes of acute cellular rejection was observed after vaccination.
Strauss et al.^38^ USA, 2021	Prospective	LT recipients 64 (48–69), (43%)	LT recipients (N= 161)	No control	Pfizer-BioNTech BNT162b2 Moderna mRNA-1273	Serological response: 130 (81%)	Not reported
Timmermann et al.^39^ Germany, 2021	Retrospective study	LT recipients 66.1, (63.6)	LT recipients (N=118)	No control	mRNA-based vaccine BNT162b2 (Pfizer) mRNA-1273 (Moderna) JNJ-78436735 (Janssen)	Seroconversion rate 118 patients (78.0%) developed anti-spike-protein-IgG antibodies	Not reported
Davidov et al.^40^ Israel, 2022	Prospective	LT recipients 59 ± 15 (56.6%) Control group 59 ± 13 (49.4%)	LT recipients (N= 76)	Immunocompetent healthcare workers (N= 174)	BioNTech BNT162b2 vaccine	A positive antibody response: LT recipients: 55 of the 76 (72.4%) Control group: 164 of the 174 (94.3%)	Liver transplant patients: The most common systemic adverse effects are fatigue and headache.
Yanis et al.^41^ USA, 2022	Prospective cohort study	SOT recipients 72.1 (61.1) Controls 62.4(34.6)	LT Patients N=20 (Cohort of 34 SOT patients)	Healthy Controls N=26	Two doses of the BNT162b2 mRNA vaccine	HCs responders 100% liver transplant (38%)	No acute cellular or antibody rejection events were noted among SOT recipients within 14 days of either vaccine dose
**Studies Focusing on Chronic Liver Disease (CLD) or Cirrhosis Only**							
Ai et al.^42^ China, 2022	Prospective, multi-center study	Cases 47.0 [38.0–56.0], (63.6) Controls 35.0 [28.5–41.5], (31.3)	CLD: N = 437	Healthy control N= 144	Inactivated whole virion SARS-CoV-2: CoronaVac, BBIBP-CorV, WIBP-CorV)	Positive rates of SARS-CoV-2 neutralizing antibodies Noncirrhotic CLD 76.8% Compensated cirrhotic group 78.9% Decompensated cirrhotic group 76.7% Healthy controls 90.3%	Most adverse reactions were mild and transient.
Bakasis et al.^43^ Greece, 2022	Prospective study	Cirrhotic 67 [27–86], (57.9%) Non-cirrhotic 65 [35–81], (42.9%) Controls 71.5 [27–88], (55%)	Cirrhotic N=38 Non-cirrhotic N=49	Controls N = 40	Pfizer-BioNTech BNT162b2 Moderna mRNA-1273	Positive rates of Anti SARS-CoV-2 antibodies: Cirrhotic 37 (97.4%) Non-cirrhotic 43 (87.8%) Controls 40 (100%)	No statistically significant difference in adverse effects between PWLD and controls. No symptomatic breakthrough infections reported in 6 month follow-up period.
Calleri et al.^44^ Italy, 2022	Prospective	Cases 56 [50–62], (69.7%) Control 55 (46–59), (70 %)	CLD pre-LT patients (N= 89)	Healthcare workers N= 30	Pfizer-BioNTech Moderna COVID-19 mRNA	Seroconversion rate Cases 84(94.4%) (after 23 days from vaccination) Control 100 % (at 4 months after vaccination)	No serious adverse effects were reported in both groups.
He et al.^45^ China, 2022	Prospective	Patients 45 (61.6) Healthy controls 44(50.6)	Chronic Hepatitis B virus infection (n = 362)	Healthy controls (n= 87)	Two doses of SARS-CoV-2 inactivated vaccine (BBIBP-CorV/CoronaVac)	% positive anti-spike Antibody After 2 months Controls 100(24/24) Cases 95.7% (112/117) At 3 months Controls 93.8% (30/32) Cases 95.5% (63/66)	The most common local and systemic AEs of CHB patients were pain (5.8%, 21/362) and fatigue (4.7%, 17/362),
John et al.^46^ USA, 2021	Retrospective	Cases 69.1 (8.4), (97.2) Control 69 (8.8), (97.2)	Liver cirrhosis (received at least 1 dose of the vaccine) (N= 20 037)	N=20 037	Pfizer BNT162b2 mRNA. Moderna mRNA-1273	Vaccine efficacy day 28 onward, % (95% CI) COVID-19 infection: 64.8 (10.9–86.1) Hospitalization for COVID: 100.0 (99.3–100.0) COVID-19–related death 100.0 (99.3–100.0)	Not reported
Wang et al.^47^ China, 2021	Prospective	Cases CLD 39.0 (33.0–48.0), (47%)	Non-alcoholic fatty liver disease (N= 381)	No control	Inactivated vaccine Beijing Institute of Biological Products Co., Ltd	Seroconversion rate 364 (95.5%)	Injection site pain, muscle pain, headache, fatigue. All adverse reactions were mild and self-limiting, and no grade 3 adverse reactions were recorded
Ai et al.^3^ China, 2022	prospective, multi-center study	CLD 47.0 (38.0–56.0), (63.6) Healthy control 35.0 (28.5–41.5), (31.3)	CLD N = 437)	Healthy control (N= 144)	Inactivated whole virion SARS-CoV-2 vaccines (600SU per dose for CoronaVac, 6.5U per dose for BBIBP-CorV and 200WU per dose for WIBP-CorV)	Positive rates of SARS-CoV-2 neutralizing antibodies Noncirrhotic CLD 76.8% Compensated cirrhotic group 78.9% Decompensated cirrhotic group 76.7% Healthy controls 90.3%	Most adverse reactions were mild and transient, and injection site pain (n=36; 8.2%) was the most frequently reported adverse event.
**Studies with Mixed Population of Both CLD/Cirrhosis and Liver Transplant (LT) Patients**							
Ruether et al.^48^ Germany, 2022	Prospective	Cases: LC patients: 53.8, (60.4%) LT recipients: 55.0, (57.2%) Controls: 50.9, (36.5%)	LT recipients (N= 138) Liver cirrhosis (N= 48)	N= 52	Pfizer BNT162b2 mRNA. OR Moderna mRNA-1273 OR AZD1222; AstraZeneca	Humoral or cellular response LT patients: 87 (63%) Liver cirrhosis: 100% Controls: 100%	Not reported
Thuluvath et al.^49^ USA, 2021	Prospective	LT Patients 65.7 ± 8.7 (66%) Cirrhosis 63.8 ± 11.1 (51%) CLD without cirrhosis 60.4 ± 13.9 (40%)	LT recipients (N= 62) Cirrhosis (N= 79) CLD without cirrhosis (N = 92)	No control	Pfizer BNT162b2 mRNA. OR Moderna OR Johnson & Johnson vaccine	Response to COVID vaccine Liver transplant Suboptimal = 27 (43.5%) Undetectable= 11 (17.8%) Optimal = 24 (39.0%) Cirrhosis Suboptimal = 15 (18.9%) Undetectable= 3 (3.7%) Optimal = 61 (77.2%) CLD without cirrhosis Suboptimal = 19 (20.6%) Undetectable= 4 (4.3%) Optimal = 69 (75%)	None of the patients who received the vaccine had any serious adverse events.

CLD, chronic liver disease; HC, healthy control; LT, liver transplant; IQR, interquartile range; SD, standard deviation; SOT, solid organ transplant.

**Table 2.** Status of Covid infection exposure, etiology of chronic liver disease (CLD), indication for liver transplantation (LT), and types of antibodies measured in the population of the included studies.

Previous COVID Infection	Etiology of CLD-cirrhosis or Indication of LT	Vaccine type/number of doses	Type of Antibody Measured	Assay of Antibody Testing
**Studies Focusing on Liver Transplant (LT) Patients Only**							
Del Bello et al.^23^ France, 2022	Not mentioned	Not mentioned	BNT162b2 vaccine [Pfizer-BioNTech]/Three doses	Anti-SARS-CoV-2 spike protein total antibodies (IgG, IgM, and IgA) (WANTAI SARS-CoV-2 Ab ELISA).	Enzyme-Linked Immunosorbent Assay (ELISA) test by Wantai (57.6%) or Other anti-SARS-CoV-2 spike protein assay (42.4%)
Boyarsky et al.^24^ USA, 2021	PCR confirmed that COVID-19 was excluded	Not mentioned	2 doses SARS-CoV-2 mRNA vaccine	Anti-spike antibody	Elecsys anti–SARS-CoV-2 S enzyme immunoassay by Roche, or EUROIMMUN enzyme immunoassay
Cholankeril et al.^25^ USA, 2022	Not mentioned	Not mentioned	2 doses of Pfizer-BioNTech (New York, NY) OR 2 doses of BNT162b2 SARS-CoV-2 vaccine	Anti-spike antibody	Electrochemiluminescence Immunoassay Analyzer (ECLIA)
Erol et al.^26^ Turkey, 2021	Prior infection with COVID-19 and acute COVID-19 infection during the study period were excluded	Not mentioned	2 vaccine doses of (Sinovac or BioNTech)	Anti-spike antibody	Quantitative assay for immunoglobulin G antibodies by Abbott
D’Offizi et al.^27^ Italy, 2021	Previous SARS-CoV2 infections were excluded.	Autoimmune (26.2%), ALD (13.8%), HBV/HCV (24.8%), NAFLD (31.3%), Others (3.9%)	2 doses of anti-SARS-CoV2 mRNA vaccines	Anti-spike IgG antibody	Chemiluminescence microparticle antibody assay (CMIA)
Guarino et al.^28^ Italy, 2021	Only patients with an adverse history of COVID-19 included	Not mentioned	2 doses of Pfizer-BioNTech BNT162b2 SARS-CoV-2 vaccination	Anti-Spike protein IgG antibody	LIAISON SARS-CoV-2 S1/S2-IgG chemiluminescent assay (DiaSorin, Italy)
Hall et al.^29^ Canada, 2021	Previous COVID Infection was Excluded	Not mentioned	2 doses of the mRNA-1273 vaccine (Moderna).	Anti-Spike protein IgG (anti-RBD) antibody	Elecsys anti–SARS–CoV–2 S enzyme immunoassay by Roche.
Herrera et al.^30^ Spain, 2021	Not mentioned	Not mentioned	2 doses of the mRNA-1273 SARS-CoV-2	Anti-spike (anti-RBD) antibody	CMIA by Atellica analyzer
Kamar et al.^31^ France, 2021	Not mentioned	Not mentioned	3 doses of the messenger RNA vaccine BNT162b2 (Pfizer–BioNTech)	Anti-spike (anti-RBD) antibody	ELISA by Wantai
Marion et al.^32^ France, 2021	Not mentioned	Not mentioned	2 doses of mRNA vaccine (BNT162b2 vaccine [Pfizer-BioNTech], or mRNA-1273 vaccine [Moderna]	Anti-spike antibody	ELISA test
Mazzola et al.^33^ France, 2021	Recent (<3 months) infection and active infection were Excluded	Not mentioned	2 doses (28 days apart) of BNT162b2 vaccine	Anti-spike RBD antibody	CMIA
Nazaruk et al.^34^ Poland, 2021	Only COVID-19 disease of more than 2 months was included	Not mentioned	2 30 μg doses of BNT162b2 vaccine	Anti-SARS-CoV-2 spike protein IgG antibody (anti-S1 antibody)	SARS-CoV-2 IgG II Quantitative test (CMIA)]
Rabinowich et al.^35^ Israel, 2021	Not mentioned	HCV (32.5%), HBV (16.2%), NASH (20), ALD (3.8%), Autoimmune (20%), Others (7.5%)	2 doses of Pfizer-BioNTech BNT162b2 SARS-CoV-2 vaccine	Anti-spike antibody	LIAISON SARS-CoV-2 S1/S2 IgG chemiluminescent assay.
Rahav et al.^36^ Israel, 2021	Patients with a positive SARS-CoV-2 PCR test before or after the first vaccination and during the first week after the second vaccination were also excluded	Not mentioned	2 doses of Pfizer-BioNTech vaccine.	Anti-spike RBD antibody	ELISA test
Rashidi-Alavijeh et al.^37^ Germany, 2021	Not mentioned	HCC (23%), PSC (16%), ALD (14%), HCV (7%), ALF (4%), WD (7%), Cryptogenic (4.7%), αAT-deficiency (4%), others (16%)	2 doses of the mRNA-based SARS-CoV-2 vaccine BNT162b2	Anti-spike antibody	ECLIA-Anti-SARS-CoV-2 IgG
Strauss et al.^38^ USA, 2021	Prior positive SARS-CoV-2 PCR was excluded	Not mentioned	2-dose mRNA vaccine series of either mRNA-1273 or BNT162b2	Anti-spike antibody	SARS-CoV-2 spike protein immunoassays (EUROIMMUN) (Indianapolis, IN)
Timmermann et al.^39^ Germany, 2021	Not mentioned	Viral hepatitis (23.7%), HCC (22%), ALD (21.1%), Autoimmune liver disease (15.3%), cryptogenic (3.4%), others (14.4%)	2 doses of mRNA-based vaccine BNT162b2 (Pfizer) 3 doses of the mRNA-based vaccine (Moderna) 1 dose of the vector-based vaccine JNJ-78436735 (Janssen Pharmaceuticals)	Anti-spike-protein-IgG antibody	Elecsys Anti-SARS-CoV-2 immune assay. Anti-SARS-CoV-2-ELISA
Davidov et al.^40^ Israel, 2022	Past or active SARS-CoV-2 infection was excluded	HCV (25.3%), NASH (17.3%), HBV (9.3%), PSC (14.7), PBC (4%), others (30.3)	3 doses of the BNT162b2mRNA	Anti-spike RBD antibody	CMIA
Yanis et al.^41^ USA, 2022	Laboratory-confirmed SARS-CoV-2 infection was excluded	Not mentioned	2 doses of the SARS-Co V-2 mRNA vaccine, BNT162b2.	Anti-spike RBD antibody	ELISA
**Studies Focusing on Chronic Liver Disease (CLD) or Cirrhosis Only**							
Ai et al.^42^ China, 2022	Active or known history of SARS-CoV-2 infection was excluded	HBV (87%), HCV (4.6%), NAFLD (2.7%), ALD (0.2%), AIH/PBC/PSC (1.8%), Others (3.7%)	2 doses of inactivated whole-virion SARS-CoV-2 vaccines.	SARS-CoV-2 neutralizing antibody	ECLIA-SARS-CoV-2 neutralizing antibody assay
Bakasis et al.^43^ Greece, 2022	Prior COVID-19 clinical infection or positive pre-vaccination anti-SARS-CoV-2 antibodies excluded	HBV (18.4%), NAFLD (23.7%), ALD (15.8%), AIH (21.1%), PBC (2.6%), PSC (7.9%), and others (10.5%)	2 doses of Pfizer-BioNTech BNT162b2 or the Moderna mRNA-1273	Anti-SARS-CoV-2 S-spike IgG antibodies and neutralizing activity	ELISA method (Euroimmun)
Calleri et al.^44^ Italy, 2022	Previous COVID-19 infection patients were not excluded	Viral hepatitis (32.6), ALD (20.2%), PBC (11.2%), Dysmetabolic cirrhosis (11.2%), others (24.8%)	2 doses of 30 mcg Comirnaty or 100 mcg of Moderna OR Single dose if previous COVID-19 infection	Anti-spike Antibody	Liaison SARS-CoV-2 TrimericS IgG assay
He et al.^45^ China, 2022	History of COVID-19 hospitalization; OR a positive SARS-CoV-2 nucleic acid test result were excluded.	Not mentioned	2 doses of SARS-CoV-2 inactivated vaccine (BBIBP-CorV/CoronaVac).	Anti-spike IgG, anti-receptor-binding domain (RBD) IgG, RBD-ACE 2 blocking antibody	Indirect ELISA method
John et al.^46^ USA, 2021	Patients with a history of COVID-19 infection were excluded	ALD (100%)	At least 1 dose of the BNT162b2 mRNA or the mRNA-1273 vaccines	Not mentioned	Not mentioned
Wang et al.^47^ China, 2021	Patients with a history of COVID-19 infection were excluded	NAFLD (100%)	2 doses of inactivated vaccine against SARS-CoV-2	Neutralizing antibodies	CLIA
Ai et al.^3^ China, 2022	Active or known history of SARS-CoV-2 infection was excluded	HBV (87.8%), HCV (4.6), NAFLD (2.7%), others (4.9%)	2 doses of inactivated whole-virion SARS-CoV-2 vaccines	Neutralizing antibodies to SARS-CoV-2	CLIA-SARS-CoV-2 neutralizing antibody assay Studies With Mixed Population of Both CLD/cirrhosis and LT patients
Ruether et al.^48^ Germany, 2022	Not mentioned	ALD (47.9), Viral (6.3%, NASH (8.3%), Autoimmune liver disease (22.9%), and others (12.6%)	2 doses of an mRNA (BNT162b2; BioNTech SE/Pfizer or mRNA-1273; Moderna Biotech) or vector-based vaccine (AZD1222; AstraZeneca)	Anti-spike Antibody	CLIA
Thuluvath et al.^49^ USA, 2021	Previous exposure to COVID-19 was excluded	HBV/HCV (36%), AIH/PBC/PSC (26%), ALD (14%), others (24 %)	2 doses of mRNA vaccines or after the single dose of the Johnson & Johnson vaccine	Antibodies to SAR-CoV-2 spike protein	Roche semi-quantitative assay (Elecsys® Anti-SARS-CoV-2 semi-quantitative)

ALD, alcoholic liver disease; αAT, α-1-ancti-trpsine; ALF, acute liver failure; CLD, AIH, autoimmune hepatitis; chronic liver disease; HBV, hepatitis B virus; HCV, hepatitis C virus; HCC. Hepatocellular carcinoma; PBC, primary biliary cirrhosis; PSC, primary sclerosing cholangitis; NAFLD, non-alcoholic fatty liver disease; NASH, non-alcoholic steatohepatitis; WD, Wilson disease.
